# COP1, the negative regulator of ETV1, influences prognosis in triple-negative breast cancer

**DOI:** 10.1186/s12885-015-1151-y

**Published:** 2015-03-15

**Authors:** Mao Ouyang, Hua Wang, Jieyi Ma, Weiming Lü, Jie Li, Chen Yao, Guangqi Chang, Jiong Bi, Shenming Wang, Wenjian Wang

**Affiliations:** 1 Laboratory of Department of Surgery, First Affiliated Hospital, Sun Yat-sen University, 58 Zhongshan Rd II, Guangzhou, Guangdong 510080 People’s Republic of China; 2 Department of Clinical Laboratory, First Affiliated Hospital, Sun Yat-sen University, 58 Zhongshan Rd II, Guangzhou, Guangdong 510080 People’s Republic of China; 3 Department of Vascular, Thyroid and Breast Surgery, First Affiliated Hospital, Sun Yat-sen University, 58 Zhongshan Rd II, Guangzhou, Guangdong 510080 People’s Republic of China

**Keywords:** ETV1, COP1, Triple-negative breast cancer, Overall survival, Prognosis

## Abstract

**Background:**

ETS variant 1 (ETV1) and E3 ubiquitin ligase constitutive photomorphogenetic 1 (COP1) have been proposed to be a pair of oncogene and tumor suppressor. However, the co-existing status of ETV1 and COP1 in triple-negative breast cancer (TNBC) and their predictive role in determining the patient’s outcome are uncertain.

**Methods:**

We examined the abundance of COP1 and ETV1 proteins and their clinicopathologic significance in archival TNBC tissues from 105 patients by tissue microarray. The potential function link between COP1 and ETV1 was observed in MDA-MB-231 cells by cell proliferation, invasion and migration assays.

**Results:**

ETV1 expression was higher in TNBC tissues compared to normal tissues, while COP1 was lower. ETV1 expression was negatively associated with COP1 abundance in TNBCs. Overexpression of COP1 led to significant reduction of ETV1 in MDA-MB-231 cells, and suppressed the cells migration and invasion. Rescue of ETV1 expression in the presence of COP1 notably regained the cells behaviors. ETV1-positive group was associated with a markedly poor overall survival. Meanwhile, we had observed favourable prognosis in COP1-positive cases for the first time. Multivariate analysis showed that COP1 together with ETV1 were independent risk factors in the prognosis of TNBC patients.

**Conclusions:**

COP1 might be a tumor suppressor by negative regulating ETV1 in patients with TNBCs. COP1 and ETV1 are a pair of independent predictors of prognosis for TNBC cases. Thus, targeting them might be a potential strategy for personalized TNBC treatment.

**Electronic supplementary material:**

The online version of this article (doi:10.1186/s12885-015-1151-y) contains supplementary material, which is available to authorized users.

## Background

Triple-negative breast cancer (TNBC) is a subtype of breast cancer defined as tumors that lack the expression of estrogen receptors (ER), progesterone receptors (PR), and HER2, and accounts for 15% of all breast cancer cases [1]. TNBC is a challenging disease with the worst outcome among all breast cancer subtypes because it does not respond to endocrine therapy or other available targeted agents [2-4]. A minority among all TNBC patients is sensitive to chemotherapy [5]. Although the metastatic potential of all subtypes of breast cancer is ultimately similar, TNBC is associated with a shorter median time of relapse and death [3,6]. Therefore, identification of the prognostic factors or markers to reliably select high and low risk subsets of TNBC cases is an urgent need for personalized therapies.

The PEA3 subfamily of ETS transcription factors is composed of ETV1, 4 and 5 [7,8]. Their association with cancer was first noted over a decade ago in Ewing tumors, in which the EWS gene can be translocated onto the three member genes and the resultant fusion proteins exerts oncogenic properties [9]. Thereafter, it was found that the PEA3 group could play a tumorigenic role in melanoma, prostate cancer, gastrointestinal stromal tumor and breast cancer [10-14]. The proposed mechanisms of breast tumorigenesis by PEA3 group partly include that they promote breast cancer incidence, progression and invasion through transcriptional activation of several genes, such as HER2, smad7, matrix metalloproteinases (MMPs) and cyclooxygenase (COX)-2 by interaction with the coactivators CBP and p300, and an associated kinase [1,15,16].

COP1 (also known as RFWD2) is a member of the COP–DET–FUS protein family. COP1 possesses E3 ubiquitin ligase activity, which is involved in the ubiquitylation of various protein substrates to trigger their proteasomal degradation [17]. As a tumor suppressor, partial or tissue-specific loss of COP1 function causally contributes to tumorigenesis [18-20]. The accumulative list of COP1 substrates identified so far includes p53, c-Jun, and PEA3 family members [21]. ETV1, ETV4 and ETV5 have highly conserved COP1-binding motifs, which can recognize COP1 [17,22]. COP1 regulates the stability and the transcriptional activity of PEA3 factors, which is dependent on the RING domain of COP1 [16]. COP1^hypo/hypo^ mice were reported to spontaneously develop malignancies at a high frequency [19]. Also, COP1 deficiency in mouse prostate elevated ETV1 and increased cell proliferation, hyperplasia, and early prostate intraepithelial neoplasia [18]. It is unclear whether dysregulation of PEA3 factors and COP1 happens concurrently in TNBCs. Therefore, it would be of great interest to investigate the potential function link between PEA3 factors and COP1, as well as the prognostic value of their expression status in TNBCs.

In the present study, we analyzed the correlation between COP1 and ETV1 expression status, and clinicopathological features, as well as the clinical outcome in a retrospective study cohort of post-resection patients with TNBC. Our data suggest that ETV1 is negatively regulated by COP1. They might be a pair of potential independent biomarkers and perhaps therapeutic targets for TNBCs.

## Methods

### Patients and tumor samples

Archived and paraffin-embedded samples were obtained from 105 TNBC patients who underwent surgical resection between January 1997 and December 2007 in the Department of Vascular, Thyroid and Breast Surgery, the First Affiliated Hospital of Sun Yat-sen University. Four pairs of fresh TNBC tissues and adjacent non-tumor tissues were obtained recently from four TNBC cases. Among all the 105 patients, the ages ranged from 24 to 82 years (mean age, 51.2 years). Ultrasonography and X-ray scans were conducted for all the patients before operation. All cases had been confirmed by hematoxylin-eosin staining and immunohistochemical detection of ER, PR and HER2 (Additional file 1: Figure S1). All patients had received anthracyclines or taxanes-based adjuvant chemotherapy after surgery dependent of age or general condition. We obtained the patients’ prior written informed consent and approval via the Institute Research Ethics Committee of the First Affiliated Hospital of Sun Yat-sen University for use of these clinical materials in this study. Most tumors (n = 93) were invasive ductal carcinomas. The remaining tumors were 3 cases of invasive lobular carcinoma, 3 cases of mucinous carcinoma, 2 cases of medullary carcinoma, 2 tubular carcinoma and 2 invasive micropapillary carcinoma. Tumor stages and clinicopathological classification were defined with the pathologic tumor-node-metastasis (pTNM) classification [23]. The overall survival was defined as the time from the date of surgery to the date of death as a result of any cause. The patients who were alive on the date of the last follow-up were censored on that date. The median duration of follow-up was 78.6 months (range, 6.0 to 145.3 months). The clinicopathologic characteristics of the patients are summarized in Table 1.

**Table 1:** **Clinicopathologic characteristics of the patients**

Factor	No.	%
**Age (years)**		
≤50	63	60
>50	42	40
**Pathological type**		
Invasive ductal carcinomas	93	88.6
Others	12	11.4
**Stage**		
I	6	5.7
II	34	32.4
III	65	61.9
**Tumor size**		
≤2 cm	19	18.1
>2 cm	86	81.9
**Relapse**		
Yes	38	36.2
No	67	63.8
**Lymph node metastasis**		
0-4	45	42.9
5-8	39	37.1
≥9	21	20.0
**Status**		
Dead	67	63.8
Alive	38	36.2

### Cell culture, plasmids, lentivirus and siRNAs

TNBC cell line, MDA-MB-231, was purchased from American Type Culture Collection (ATCC). The cells were cultured using DMEM (Gibco, USA) supplemented with 10% fetal bovine serum (Gibco, USA) at 37°C and 5% CO_2_. Full-length human ETV1 coding sequence was cloned into GV141 vector (GV141-ETV1) and lentivirus containing full-length human COP1 coding sequence and GFP (LVCOP1) was constructed using GV287 vector and packed by Genechem (Genechem Co., China). Lentivirus with empty vector was used as negative control (LV-NC). siRNAs against ETV1 or COP1 (Additional file 2: Table S1) and negative control siRNAs were purchased from Ribobio (Ribobio Co., China). Transfection efficiency of MDA-MB-231 cells was detected by qRT-PCR assay and western blotting (Additional file 3: Figure S2, Additional file 4: Figure S3 and Additional file 5: Figure S4).

### Cell proliferation assay

To determine the effect of enforced COP1 expression and suppression of ETV1 on cell proliferation, MDA-MB-231 cells were infected with 1 × 10^8^TU/ml LV-NC or LVCOP1 in enhanced infection solution containing 5 μg/μl polybrene (Genechem Co., China) and screened by flow cytometry to obtained positive infected cell lines to expand culture for further investigation. For cell proliferation assay, MDA-MB-231 and lentiviruses infected cells were seeded at a density of 3000 cells/well in 96 well plate. Then, siRNAs and GV141-ETV1 were transfected into cells with Lipofectamine 2000 (Invitrogen, USA) according to the manufacture’s instruction. After 72 h, cells were subjected to cell counting kit-8 (CCK8) assay. Cell proliferation was determined by reading plates at 450 nm.

### Cell invasion and migration assays

The cancer cell invasion assay was performed in BD BioCoat™ Matrigel™ Invasion Chamber (8 μm pore size) (BD Bioscience, USA) and transwell migration assay was performed in cell culture inserts with PET membrane (Corning, USA). Assays were performed as instructed by the manufacturer’s protocols. Briefly, cells (1 × 10^5^ cells) suspended in serum free medium were added to the upper chamber of an insert, and the insert was placed in a 24-well plate with DMEM supplemented with 10% fetal bovine serum. Migration assays were carried out for 6 h and invasion assays were carried out for 16 h. After the cells were incubated for exact time at 37°C, the inserts were washed with PBS, and cells on the upper surface of the insert were removed with a cotton swab. Cells adhering to the lower surface were fixed with 4% formaldehyde for 30 minutes, stained with 0.1% crystal violet solution and captured under a microscope.

### Total RNA isolation and qRT-PCR assay

The samples were stored in liquid nitrogen until RNA extraction. The total RNA from each sample was extracted using Trizol reagent (Invitrogen, USA) according to the manufacturer’s instruction. 2 μg RNA from each sample was used to synthesize the first-strand cDNA using the PrimeScript™ II 1st Strand cDNA Synthesis Kit (TaKaRa, China) according to the manufacture’s instruction. qRT-PCR assays were then performed on CFX96 (Bio-Rad Laboratories, USA) utilizing SYBR® Premix Ex Taq™ II (Tli RNaseH Plus) (TaKaRa, China). To normalize the target gene’s expression levels in different groups, β-actin was chosen as an internal control. Primer sequences used in this study were listed in Additional file 6: Table S2. The relative mRNA expression levels of target genes were calculated using the 2^-ΔΔCt^ method.

### Western blotting analysis

To perform western blotting analysis, the samples were lysed in RIPA protein lysis buffer (Beyotime, China) supplemented with 1 mM PMSF. Protein concentration was measured by BCA protein assay kit (Beyotime, China). 20 μg of target protein was applied to each well of 10% SDS-polyacrylamide gel (SDS-PAGE), separated electrophoretically and transferred onto polyvinylidene difluoride membranes (Millipore Corporation, USA). The transblotted membrane was incubated with anti-ETV1 rabbit polyclonal antibodies (1:1000; ABCAM, USA) and anti-COP1 mouse monoclonal antibody (1:1000; ABCAM, USA) at 4°C overnight, respectively. Expression of ETV1 or COP1 was detected by horseradish peroxidase (HRP) conjugated secondary antibody (1:5000; CST, USA) and an enhanced chemiluminescence kit (Amersham Pharmacia Biotech, UK) according to the manufacturer suggested protocols. An anti-GAPDH mouse monoclonal antibody (1:2000; Kangcheng, China) was used as a loading control.

### Tissue microarray preparation

For tissue microarray block construction, a hematoxylin and eosin section from formalin-fixed and paraffin-embedded tumor tissues was reviewed to confirm the diagnosis and to define representative tumor regions. Thereafter, two 1 mm-sized core biopsies were taken from the morphologically representative areas using a precision instrument (MicroDigital Co., Korea) and then arrayed onto a recipient paraffin block. The core biopsies were reviewed by two pathologists. In total, 2 tissue microarray blocks were constructed from 105 TNBC cases. Each of the recipient blocks included one core of normal breast tissue for internal control, and two cores of palatine tonsil and placenta for proper tissue array orientation and universal control. After construction, two 4 μm sections were removed from each block and transferred onto glass slides for immunohistochemical staining. The quality of morphologic preservation in the tissue array sections were assessed against the whole tissue sections of the same cases. A valid case was defined as a tumor occupying more than 10% of the core area [24].

### Immunohistochemical analysis

Immunohistochemical staining of ETV1 or COP1 was carried out according to the manufacture’s protocol on all four TNBC tissue microarrays, respectively. In briefly, the sections were incubated overnight in a moist box with antibodies of ETV1 (1:100; ABCAM, USA) or COP1 (1:200; ABCAM, USA) in PBS at 4°C. Poly peroxidase-anti-mouse/rabbit IgG (GSGB-BIO, China) was subjected to the sections for 30 minutes at room temperature after washing with PBS. Diamindobenzidine was used for colorimetric detection and the sections were counterstained with haematoxylin and mounted with distyrene plasticizer xylene (DPX). Negative controls were performed by replacing the primary antibody with preimmune rabbit serum. Positive controls were conducted according to the manufacture’s suggestion. For each run of immunohistochemistry, negative and positive controls were performed.

Immunostained tissue array sections were reviewed under a microscope by two pathologists, who were blinded regarding the clinicopathological characteristics and outcome of the patients, while visually scoring each individual tissue core. An immunoreactivity score method based on the proportion and intensity of positively stained tumor cells was employed. For positively stained cells: 0 (negative) was denoted for < 10% positive cells, 1 (weak) for < 25% positive cells, 2 (moderate) for < 50% positive cells, and 3 (strong) for > 50% positive cells. The staining intensity was defined as: 0 for no stain, 1 for weak-positive (faint yellow), 2 for moderate-positive (yellowish-brown), and 3 for strong-positive (brown). Scores of the proportion and intensity of positively stained tumor cells were added and stratified as having negative (−) expression (0–3 score) and positive (+) expression (4–6 score) [25]. If no tumor was detected, or staining could not be evaluated for all four cores, the status of ETV1 or COP1 was noted as missing.

### Statistical analysis

All statistical analyses were carried out using the SPSS 16.0 statistical software package. The *χ*^2^ test or Fisher’s exact test were used to analyze the relationship between ETV1 and COP1 expression and clinicopathological parameters. Survival curves were evaluated by the Kaplan-Meier method and compared using the log-rank test between the positive and negative expression of ETV1 or COP1 cases. Cox proportional hazard regression models were used to evaluate the association of ETV1 and COP1 status with survival outcomes after adjusting for covariates. Forward: LR multivariate Cox regression analysis was used to find independent prognostic factors. All statistical tests were two sided and *P* < 0.05 was considered statistically significant.

## Results

### The expression level of ETV1 is inverse with COP1 in clinical TNBC samples

**Figure 1:**
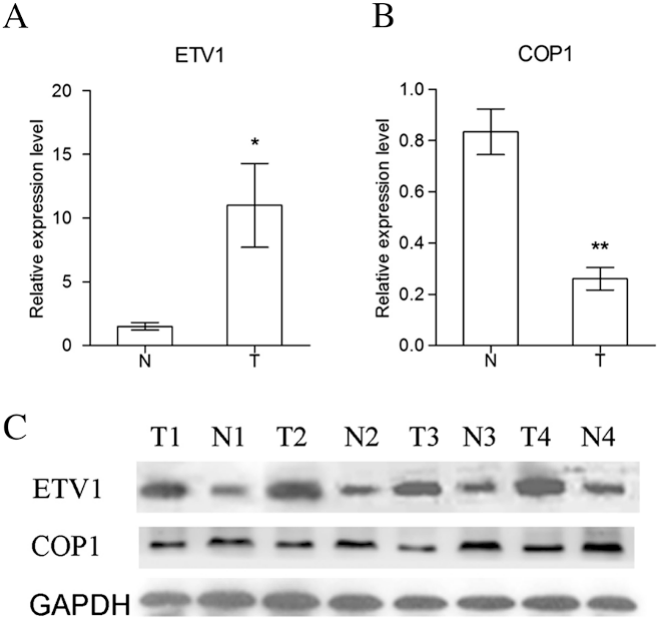
**ETV1 and COP1 expression levels in four pairs of tumor (T) and normal (N) breast tissues were detected by qRT-PCR (A, B) and western blotting (C).** Data represented relative mRNA expression levels (mean ± SEM) to one of the normal samples. (Students t tests, *P < 0.05; **P < 0.01).

We utilized qRT-PCR and western blotting to quantify the expression levels of both ETV1 and COP1 in TNBC tissues. Our results showed that the expression levels of ETV1 mRNA and protein in TNBC tissues (T) were significantly higher than those of the paired adjacent non-cancerous tissues (N). Whereas, expression levels of COP1 mRNA and protein in TNBC tissues (T) were markedly lower than those of the paired adjacent non-cancerous tissues (N) (Figure 1). Our data revealed that TNBC tissues with relatively higher ETV1 expression displayed much less COP1 abundance, indicating that there might be an inverse relationship between ETV1 and COP1 in TNBC.

### The expression status of ETV1 and COP1 is related with clinical factors and prognosis of TNBC patients

105 TNBC patients were involved in this retrospective study. The median age at breast cancer diagnosis was 50 years old (range, 24–82 years). The median duration of follow-up after surgery was 78.57 months (range, 6.0-145.3 months), with 67 deaths at the end of follow up.

**Figure 2:**
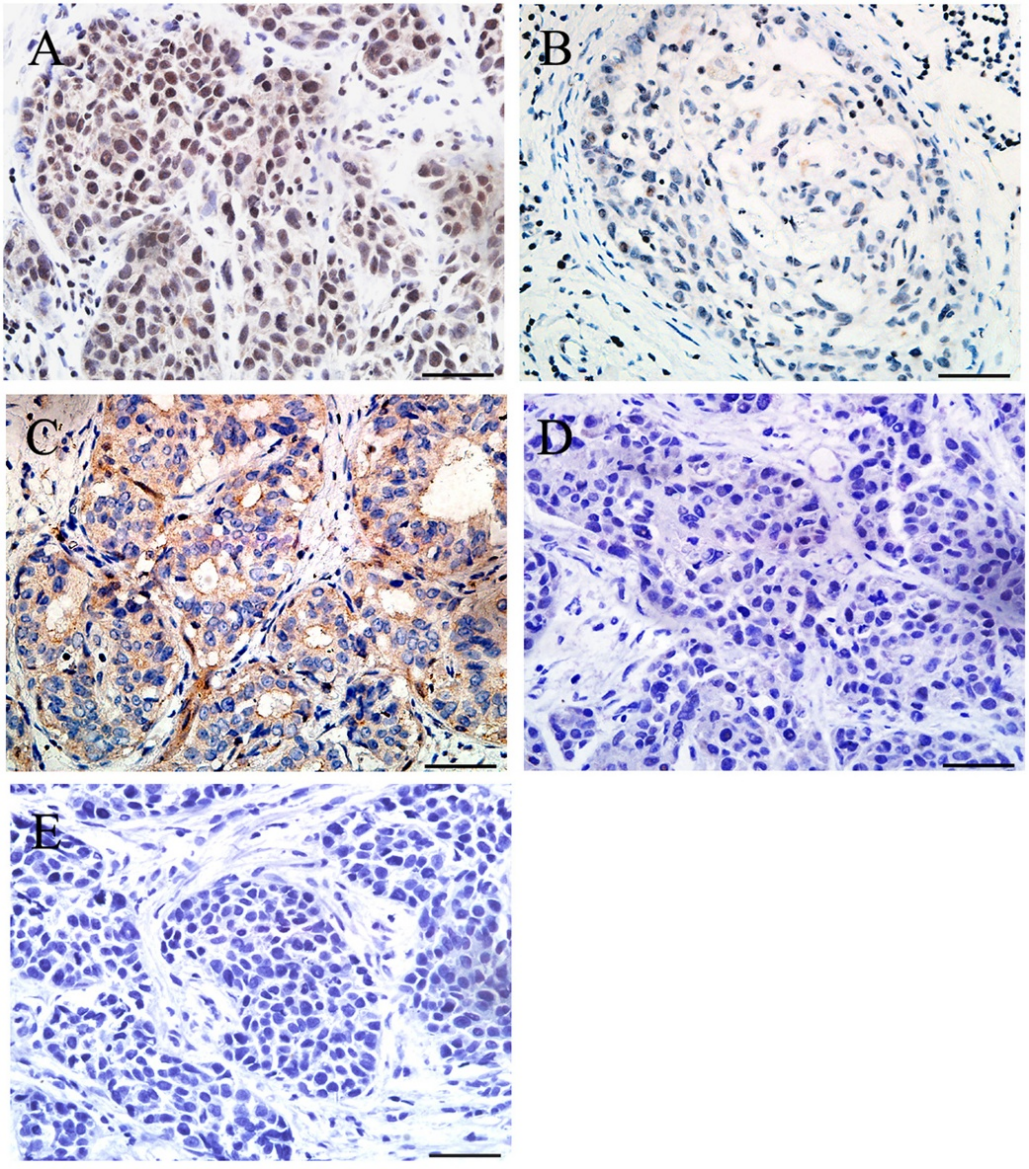
**Immunohistochemical staining of ETV1 and COP1 in human TNBC tissues. A**, Positive expression of ETV1 in TNBC. **B**, Negative expression of ETV1 in TNBC. **C**, Positive expression of COP1 in TNBC. **D**, Negative expression of COP1 in TNBC. **E**, Negative control of ETV1 or COP1 in TNBC. Scale bar, 50 μm.

Among the 105 cases of TNBC, 79 cases (75.2%) were ETV1-positive and 26 (24.7%) were ETV1-negative, whereas 30 cases (28.6%) were COP1-positive and 75 were negative (Figure 2). ETV1 expression status according to COP1 expression and their corresponding clinicopathological characteristics are summarized in Table 2. There was a significant negative correlation between ETV1 and COP1 expression (*χ*^2^ test, γ = − 0.960, *χ*^2^ = 52.678; *P* < 0.001), indicating potential interaction between the two proteins. Either ETV1 or COP1 expression status was significantly associated with TNM stages of TNBC, the number of lymph nodes involved and tumor relapse. Age, tumor size and pathological types had not been found to be associated with ETV1 or COP1 expression status. These results suggest that both ETV1 and COP1 were aberrantly expressed in TNBC tissues, and might influence the outcomes of patients.

**Table 2:** **Comparison between ETV1 and COP1 expression, and clinicopathologic parameters in 105 TNBC cases**

Characteristics	ETV1		*χ* ^2^	*P*value	COP1		*χ* ^2^	*P*value
	Negative	Positive			Negative	Positive		
**Age (y)**								
≤50	14	49	0.545	0.460	41	17	0.213	0.645
>50	12	30			35	12		
**Tumor Size**								
≤2 cm	5	14	−0.030	0.535^▲^	13	6	0.182	0.443
>2 cm	21	65			63	23		
**Pathological type**								
Invasive ductal carcinomas	21	72	−2.078	0.139^▲^	69	24	1.337	0.204
Others	5	7			7	5		
**TNM Stage**								
I	3	3	−6.220	0.045^▲*^	2	4	−7.466	0.024^▲*^
II	12	22			22	12		
III	11	54			52	13		
**Lymph node metastasis**								
0-4	17	28	−16.986	0.028^▲*^	31	22	−10.405	0.006^▲*^
5-8	6	33			29	5		
≥9	3	18			16	2		
**Relapse**								
No	23	44	9.094	0.002	41	26	−11.590	0.000^▲*^
Yes	3	35			35	3		

*means *P* < 0.05; − means *χ*^2^ of Fisher exact test; ^▲^means *P* value of Fisher exact test.

**Figure 3:**
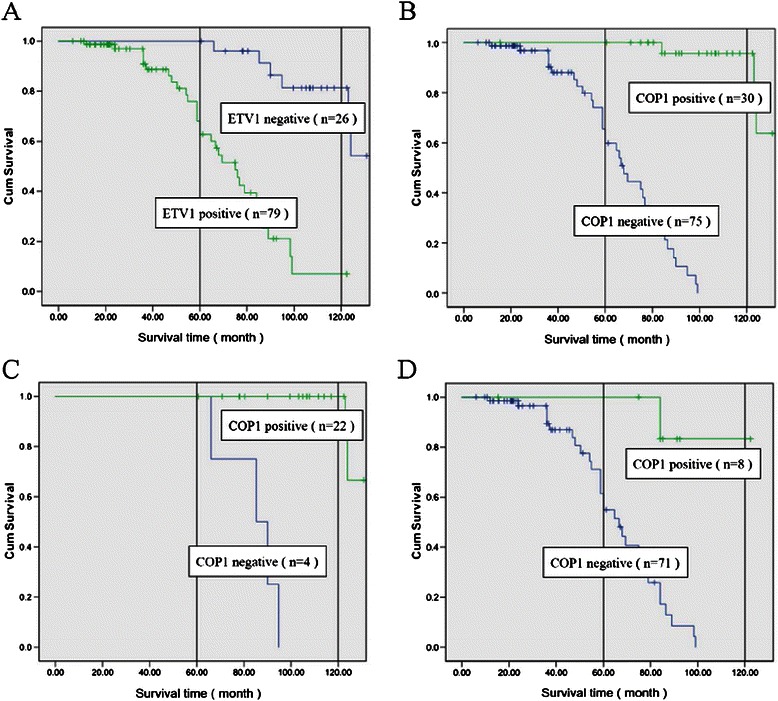
**Overall survival of patients with TNBCs according to ETV1 and COP1 protein expression statuses. A**, Significant difference in overall survival time was observed between ETV1-positive and ETV1-negative group (*P* < 0.001). **B**, COP1-positive TNBC patients had longer overall survival time than that of COP1-negative TNBC patients (*P* < 0.001). **C**, In 26 cases of ETV1-negative TNBCs, COP1-positive cases had significant higher survival rate than that of COP1-negative group (*P* < 0.001). **D**, In 79 cases of ETV1-positive TNBCs, COP1-positive cases had significant higher survival rate than that of COP1-negative group (*P* < 0.001).

Then, the proteins expression status of ETV1 or COP1 in TNBC tissues were investigated for associations with overall survival using Kaplan–Meier analysis, and log-rank test for significance estimates. Figure 3A showed that women with ETV1-positive tumors had a shorter overall survival time than women with ETV1-negative tumors (log-rank test, *P* < 0.05). However, compared to tumors with negative COP1 expression, positive COP1 expression was correlated with a significant decrease of breast cancer overall mortality during the follow-up period (log-rank test, *P* < 0.001) (Figure 3B). Among women with ETV1-positive tumors, the estimated 5-year and 10-year TNBC specific overall survival was 62.8% and 7.0%, respectively, whereas among women with ETV1-negative tumors, the rates were 100% and 81.3%, respectively (Figure 3A). Meanwhile, among women with COP1-positive tumors, the estimated 5-year and 10-year TNBC specific overall survival was 100% and 95.7%, respectively, and for women with COP1-negative tumors, the rates were 59.8% and 0%, respectively (Figure 3B). In 79 ETV1-positive tumors, COP1-positive cases showed significantly higher survival rate than that of patients in the COP1-negative group (log-rank test, *P* < 0.001) (Figure 3C and D). The Kaplan–Meier curves and log-rank test showed that women with ETV1-negative/COP1-positive tumors had the best survival relative to women with the other subtypes (log-rank test, *P* < 0.001) (Figure 3C and D). These results indicate that ETV1 is a poor prognostic factor, and COP1 is a favourable prognostic factor for TNBCs.

In multivariate Cox regression analysis, the Cox proportional hazards model was adjusted for age, pathological type, TNM stage, number of lymph node metastasis, relapse, and tumor size. The results (Table 3) showed that the COP1 and ETV1 expression status, and TNM stage were significantly associated with patients overall survival. However, patients’ age, the number of lymph nodes involved, tumor size and tumor relapse were not significantly associated with overall survival. Furthermore, forward: LR multivariate Cox regression analysis confirmed that ETV1 (RR = 1.502; 95% CI: 1.100 – 2.044; *P* < 0.05) and COP1 (RR = 0.650; 95% CI: 0.149 – 6.732; *P* < 0.001) expression status were independent prognostic predictors of overall survival of TNBC patients.

**Table 3:** **Multivariate analysis of patients’ survival**

	Regression coefficient	SE	Relative risk (RR)	95.0% CI for RR		*P*
				Lower	Upper	
TNM stage	0.514	0.088	1.672	1.407	1.987	0.009*
Relapse	−0.780	0.586	0.458	0.145	1.446	0.183
Lymph node	0.434	0.406	1.544	0.696	3.425	0.285
ETV1	0.405	0.158	1.502	1.100	2.044	0.028*
COP1	−0.431	0.753	0.650	0.149	6.732	0.000*
Age	0.291	0.268	1.338	0.792	2.261	0.276
Tumor size	0.449	0.610	1.566	0.474	5.175	0.462
Pathological type	0.857	0.677	2.357	0.626	8.879	0.205

*For ETV1 or COP1, positive was labeled as 1, and negative was labeled as 0. Stage I, II, III were labeled as 1, 2, 3, respectively.

### COP1 might suppress TNBC cell invasion, migration by targeting ETV1

**Figure 4:**
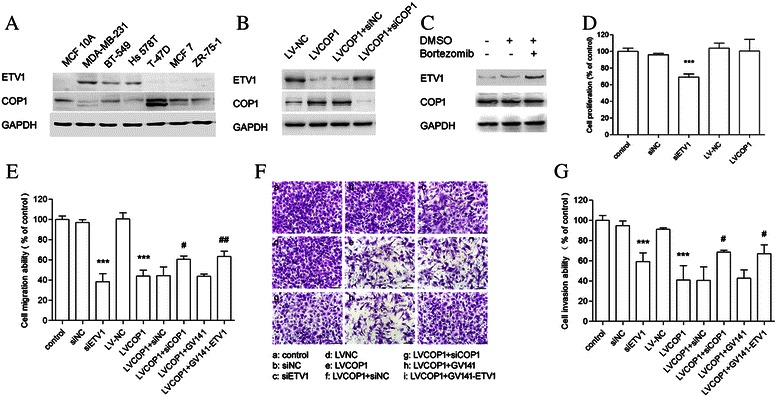
**The potential function link between COP1 and ETV1 in MDA-MB-231 cells. A**, Expression of ETV1 and COP1 in breast cancer cell lines. **B**, MDA-MB-231 cells were infected with recombinant COP1 lentivirus (LV-COP1) or lentivirus with empty vector (LV-NC). LV-COP1 infected cells were further transfected with siCOP1, a non-specific RNA (siNC) as a negative control. Western blotting showed inverse relationship between COP1 and ETV1 expression. **C**, LVCOP1 infected MDA-MB-231 cells were treated with DMSO or 2 μM Bortezomib for 2 h and then subjected to western blotting. **D**, MDA-MB-231 cells were transfected with siETV1 or siNC, or were infected with LV-NC or LV-COP1 for 72 h. Normal MDA-MB-231 cells were considered to be control group. CCK8 assay showed that siETV1 significantly decreased the cell proliferation while overexpression of COP1 did not influence the cell proliferation of MDA-MB-231. **E**-**G**, MDA-MB-231 cells were treated as showed in the figures and cultured for 48 h. Cell migration and invasion assays were performed using cell culture inserts with PET membrane and BD matrigel invasion chamber, respectively. Results indicated that suppressing ETV1 or up-regulating COP1 decreased the migration **(E)** and invasion **(F, G)** ability of MDA-MB-231 cells. Turnover ETV1 expression in LV-COP1 infected cells partly rescued the migration and invasion function of cells. Scale bar, 100 μm. (Students t tests, ***P < 0.001 versus MDA-MB-231 control group; #*P* < 0.05, ##*P* < 0.01, versus LV-COP1 group).

To further study the effect of COP1 on ETV1, we carried out the trials in a TNBC strain, MDA-MB-231 cell line. First of all, we observed COP1 and ETV1 expression status in several breast cancer cell lines, and found that breast cancer cell lines with relatively higher ETV1 expression displayed less COP1 abundance (Figure 4A). We found that MDA-MB-231 cell had high expression level of ETV1 and low expression of COP1. Thereafter, we chose MDA-MB-231 cell line to perform further investigation. Our results showed that there was an inverse relationship between COP1 and ETV1 expression. Overexpression of COP1 significantly decreased ETV1 protein level. COP1 knock-down or proteasome inhibition with Bortezomib in the presence of COP1 notably increased ETV1 expression (Figure 4B and C). CCK8 assay showed that siETV1 markedly decreased the cell proliferation while overexpression of COP1 did not influence the cell proliferation of MDA-MB-231 (Figure 4D). To substantiate the potential interaction between COP1 and ETV1, we performed cancer cell invasion assays and transwell assays using MDA-MB-231 cells. Results indicated that suppressing ETV1 expression or up-regulating COP1 decreased the migration (Figure 4E) and invasion (Figure 4F and G) ability of MDA-MB-231 cells. Rescue of ETV1 expression in the presence of COP1 significantly recovered the invasion and migration capacity of MDA-MB-231 cells. These results suggested that COP1 might suppress TNBC cell invasion and migration by targeting ETV1.

## Discussion

ETV1 (also called ER81), is an oncogene for a variety of human malignant diseases. Several lines of evidence showed that ETV1 is aberrantly overexpressed in triple-negative breast tumor cells or tissues, which presented the causative role of ETV1 in breast tumorigenesis [13,26,27]. Our results revealed that ETV1 was predominantly overexpressed in TNBC tissues. Knock-down ETV1 could significantly depress invasion, migration and proliferation behavior of MDA-MB-231 cells. These data are in good accordance with the previous studies [13,26]. We found that ETV1-positive expression was significantly correlated with TNM stages of TNBCs, lymph node metastasis and tumor relapse. Furthermore, our results showed that women with ETV1-positive tumors had shorter survival times than women with ETV1-negative tumors during the period of follow-up, indicating that ETV1 might be a poor predictor of TNBC prognosis. Multivariate analysis revealed that ETV1 was independent risk factor in predicting prognosis of TNBC patients. These results support an earlier hypothesis that dysregulation of ETV1 might be causally involved in breast tumorigenesis [27,28]. Of note, it is well established that activation of ETV1 is regulated by HER2/KIT-RAS-RAF-MEK-MAPK pathway [7,12,29]. However, TNBC is lack of HER2. Whether transcriptional activation of ETV1 in TNBCs is stimulated by KIT-RAS-RAF-MEK-MAPK or other pathways needs to be further validated.

COP1 has been confirmed as a tumor suppressor in series cancer diseases [18-20]. However, there is no report of COP1 referred to clinical TNBC cases. In our study we found that COP1 expression in TNBC tissues was obviously lower than that of normal breast tissues. COP1 expression status was associated with TNM stages of TNBCs, lymph node metastasis and tumor relapse. Our data showed that overexpression of COP1 could depress invasion and migration capacity of MDA-MB-231 cells. Moreover, patients with COP1-positive tumors had longer survival times than COP1-negative cases, indicating that COP1 might be a favourable predictor of TNBC prognosis. Furthermore, multivariate analysis demonstrated that COP1 was independent risk factor in predicting prognosis of the patients. These results suggest that decrease of COP1 might be causatively involved in TNBC tumorigenesis.

ETV1 is known to be degraded after being ubiquitinated by COP1 [17]. Therefore, loss of COP1 expression or COP1 deficiency may be an important mechanism that leads to overexpression of ETV1 in cancer, promoting tumorigenesis [18,22]. Truncated ETV1 lacking the COP1 binding motifs were shown to be 50-fold more stable than the wild-type ETV1. In addition, COP1 deficiency increased cell proliferation, hyperplasia, and early prostate intraepithelial neoplasia by elevating ETV1 expression in mouse prostate [18]. In the present study, we found that ETV1 abundance was inversely associated with COP1 expression status, which is consistent with the previous reports [18,22]. To confirm their potential function link, we constructed COP1 overexpression MDA-MB-231 strains by lentivirus infection. The results revealed that up-regulation of COP1 led to significant reduction of ETV1 in MDA-MB-231 cells, and refrained the cells invasion and migration capacity. Turnover ETV1 abundance in recombinant COP1 lentivirus infected cells partly rescued the migration and invasion behaviours of the cells. These results suggested that COP1 might suppress TNBC cell invasion and migration by regulating ETV1. Escape of ETV1 from COP1-mediated degradation could underlie breast tumorigenesis [18,22]. Moreover, it has been confirmed that COP1 has other substrates besides ETV1 [17,18]. As a substrate of COP1, c-Jun was found to be constitutively kept at low levels by COP1. Furthermore, COP1 deficiency stimulated cell proliferation in a c-Jun–dependent manner [19]. When expressed as a mutant, COP1 could no longer interact with c-Jun [30]. In our study, among ETV1-negative cases, women with COP1-positive tumors had the most favourable survival compared to women with the other subtypes. The results support the hypothesis that COP1 might play a role in tumor suppression by degrading or ubiquitinating multiple substrates, besides ETV1. A conflicting report showed that COP1 acted as an E3 ubiquitin ligase for p53 and inhibited p53-dependent transcription and apoptosis [31,32]. However, a recent study did not show increase of p53 abundance and transcriptional activity in cells or tissues from COP1-deficient mice [19]. Whether there are other interactive molecules of COP1, and if so, their functional or pathogenic role in TNBC occurrence needs further investigation.

This study had several limitations. First, it was a retrospective study. Second, we did not employ an animal model to confirm that COP1 might suppress TNBC cell invasion and migration by targeting ETV1.

## Conclusions

In conclusion, we demonstrated that COP1 might be a tumor suppressor by negatively regulating ETV1 in patients with TNBC. For the first time we observed a favourable prognosis in COP1-positive cases from a cohort of TNBC patients. Our data suggest that COP1 and ETV1 may function as a pair of valuable independent prognostic biomarkers for TNBCs. Whether ETV1 and COP1 might be effective therapeutic targets towards changing TNBC patient outcome is worth to be explored. Further investigation is essential to determine the concurrent status of COP1 and the other two PEA3 factors in TNBCs.

## Additional files

Additional file 1: Figure S1. Microphotographs of a representative breast cancer case diagnosed as triple-negative. **(A)** Hematoxylin-Eosin staining. **(B–D)** Immunohistochemical detection for ER, PR and HER-2, respectively. It is obviously that this case is negative for ER, PR and HER-2. Scale bar, 50 μm.
Additional file 2: Table S1. siRNA sequences used in this article. The non-specific RNA (siNC) severed as a negative control are purchased from Ribobio (Ribobio Co., China).
Additional file 3: Figure S2. Estimation of recombinant COP1 lentivirus (LVCOP1) infection efficiency in MDA-MB-231 cells. **(A)** LVCOP1 infected MDA-MB-231 was revealed by fluorescence microscopy via detecting the expression of GFP. **(B)** The same location of Figure 1A was observed under phase-contrast microscopy. **(C)** qRT-PCR and western blotting showed that COP1 expression was significantly increased in MDA-MB-231 cells infected with LVCOP1. Scale bar, 100 μm. (Students t tests, ****P* < 0.001 versus MDA-MB-231 control group).
Additional file 4: Figure S3. Detection of the efficiency of ETV1 siRNA in MDA-MB-231 cells. Cells were transfected with siRNA against ETV1 or a non-specific RNA (siNC) as a negative control. The efficiency of siRNA was analyzed by qRT-PCR **(A)** and western blotting **(B)**. Results showed that siETV1 could significantly decreased the expression of ETV1. (Students t tests, **P* < 0.001 versus MDA-MB-231 control group).
Additional file 5: Figure S4. Detection of the transfection efficiency of ETV1 expression vector in MDA-MB-231 cells. Full-length human ETV1 coding sequence was cloned into GV141 vector (GV141-ETV1). Cells were transfected with ETV1 expression vector or an empty GV141 vector. The efficiency of transfection was detected by qRT-PCR **(A)** and western blotting **(B)**. Results showed that the expression of ETV1 was significantly up-regulated by GV141-ETV1. (Students t tests, ***P* < 0.05 versus control group).
Additional file 6: Table S2. Primer sequences used in this article.

## Abbreviations

COP1: Constitutive photomorphogenic 1
ETV1: ETS variant 1
TNBC: Triple-negative breast carcinoma
TNM: Tumor-node-metastasis
RR: Relative risk
CI: Confidence interval

## References

[1] Maegawa ROB, Tang S-C (2010). Triple-negative breast cancer: unique biology and its management. Cancer Investig.

[2] Foulkes WD, Smith IE, Reis-Filho JS (2010). Triple-negative breast cancer. N Engl J Med.

[3] Hudis CA, Gianni L (2011). Triple-negative breast cancer: an unmet medical need. Oncologist.

[4] Ismail-Khan R, Bui MM (2010). A review of triple-negative breast cancer. Cancer Control.

[5] Podo F, Buydens LMC, Degani H, Hilhorst R, Klipp E, Gribbestad IS (2010). Triple-negative breast cancer: present challenges and new perspectives. Mol Oncol.

[6] Cheang MCU, Voduc D, Bajdik C, Leung S, McKinney S, Chia SK (2008). Basal-like breast cancer defined by five biomarkers has superior prognostic value than triple-negative phenotype. Clin Cancer Res.

[7] Oh S, Shin S, Janknecht R (1826). ETV1, 4 and 5: an oncogenic subfamily of ETS transcription factors. Biochim Biophys Acta.

[8] Brown TA, McKnight SL (1992). Specificities of protein–protein and protein–DNA interaction of GABP alpha and two newly defined ets-related proteins. Genes Dev.

[9] Jeon IS, Davis JN, Braun BS, Sublett JE, Roussel MF, Denny CT (1995). A variant Ewing's sarcoma translocation (7;22) fuses the EWS gene to the ETS gene ETV1. Oncogene.

[10] Jané-Valbuena J, Widlund HR, Perner S, Johnson LA, Dibner AC, Lin WM (2010). An oncogenic role for ETV1 in melanoma. Cancer Res.

[11] Tomlins SA, Rhodes DR, Perner S, Dhanasekaran SM, Mehra R, Sun XW (2005). Recurrent fusion of TMPRSS2 and ETS transcription factor genes in prostate cancer. Science.

[12] Chi P, Chen Y, Zhang L, Guo X, Wongvipat J, Shamu T (2010). ETV1 is a lineage survival factor that cooperates with KIT in gastrointestinal stromal tumours. Nature.

[13] Wang Y, Wang L, Chen Y, Li L, Yang X, Li B, Song S, Yang L, Hao Y, Yang J (2011). ER81 expression in breast cancers and hyperplasia. Patholog Res Int.

[14] Yun Z, Dai T, Wang S, Peng R, Li X, Qin T (2014). Overexpression of ETV4 protein in triple-negative breast cancer is associated with a higher risk of distant metastasis. OncoTargets Ther.

[15] Dowdy SC, Mariani A, Janknecht R (2003). HER2/Neu- and TAK1-mediated up-regulation of the transforming growth factor beta inhibitor Smad7 via the ETS protein ER81. J Biol Chem.

[16] Papoutsopoulou S, Janknecht R (2000). Phosphorylation of ETS transcription factor ER81 in a complex with its coactivators CREB-binding protein and p300. Mol Cell Biol.

[17] Marine JC (2012). Spotlight on the role of COP1 in tumorigenesis. Nat Rev Cancer.

[18] Vitari A, Leong KG, Newton K, Yee C, O’Rourke K, Liu J (2011). COP1 is a tumour suppressor that causes degradation of ETS transcription factors. Nature.

[19] Migliorini D, Bogaerts S, Defever D, Vyas R, Denecker G, Radaelli E (2011). Cop1 constitutively regulates c-Jun protein stability and functions as a tumor suppressor in mice. J Clin Invest.

[20] Shao J, Teng Y, Padia R, Hong S, Noh H, Xie X (2013). COP1 and GSK3β cooperate to promote c-Jun degradation and inhibit breast cancer cell tumorigenesis. Neoplasia.

[21] Wei W, Kaelin WG (2011). Good COP1 or bad COP1? In vivo veritas. J Clin Invest.

[22] Baert JL, Monte D, Verreman K, Degerny C, Coutte L, de Launoit Y (2010). The E3 ubiquitin ligase complex component COP1 regulates PEA3 group member stability and transcriptional activity. Oncogene.

[23] Zurrida S, Veronesi U (2011). A new TNM classification for breast cancer to meet the demands of he present and the challenges of the future. Women's Health.

[24] Lee HS, Cho SB, Lee HE, Kim MA, Kim JH, Park do J (2007). Protein expression profiling and molecular classification of gastric cancer by the tissue array method. Clin Cancer Res.

[25] Sun B, Zhang S, Zhang D, Li Y, Zhao X, Luo Y (2008). Identification of metastasis-related proteins and their clinical relevance to triple-negative human breast cancer. Clin Cancer Res.

[26] Baert J-L, Monte D, Musgrove EA, Albagli O, Sutherland RL, Launoit Y (1997). Expression of the PEA3 group of ETS-related transcription factors in human breast-cancer cells. Int J Cancer.

[27] Trimble MS, Xin JH, Guy CT, Muller WJ, Hassell JA (1993). PEA3 is overexpressed in mouse metastatic mammary adenocarcinomas. Oncogene.

[28] Shin S, Bosc DG, Ingle JN, Spelsberg TC, Janknecht R (2008). Rcl is a novel ETV1/ER81 target gene upregulated in breast tumors. J Cell Biochem.

[29] Holbro T, Civenni G, Hynes NE (2003). The ErbB receptors and their role in cancer progression. Exp Cell Res.

[30] Wertz IE, O’Rourke KM, Zhang Z, Dornan D, Arnott D, Deshaies RJ (2004). Human De-etiolated-1 regulates c-Jun by assembling a CUL4A ubiquitin ligase. Science.

[31] Dornan D, Bheddah S, Newton K, Ince W, Frantz GD, Dowd P (2004). COP1, the negative regulator of p53, is overexpressed in breast and ovarian adenocarcinomas. Cancer Res.

[32] Dornan D, Wertz I, Shimizu H, Arnott D, Frantz GD, Dowd P (2004). The ubiquitin ligase COP1 is a critical negative regulator of p53. Nature.

