# Supplementary figures and images for: COP1, the negative regulator of ETV1, influences prognosis in triple-negative breast cancer
Source: BMC Cancer. 2015 Mar 15;15:132. doi: 10.1186/s12885-015-1151-y (PMC4381371; doi:10.1186/s12885-015-1151-y)

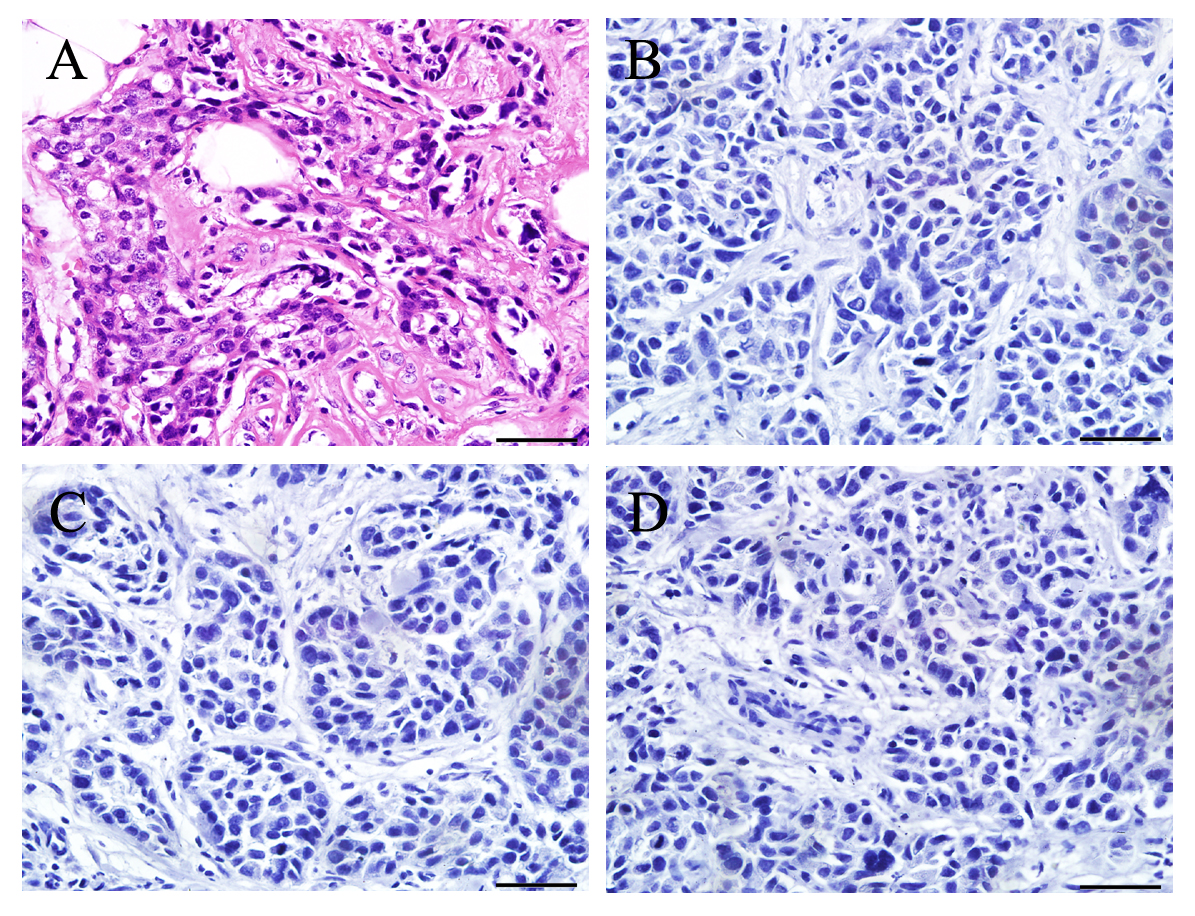

Supplement: Additional file 1: Figure S1. — Microphotographs of a representative breast cancer case diagnosed as triple-negative. (A) Hematoxylin-Eosin staining. (B–D) Immunohistochemical detection for ER, PR and HER-2, respectively. It is obviously that this case is negative for ER, PR and HER-2. Scale bar, 50 μm. [file 12885_2015_1151_MOESM1_ESM.tiff]

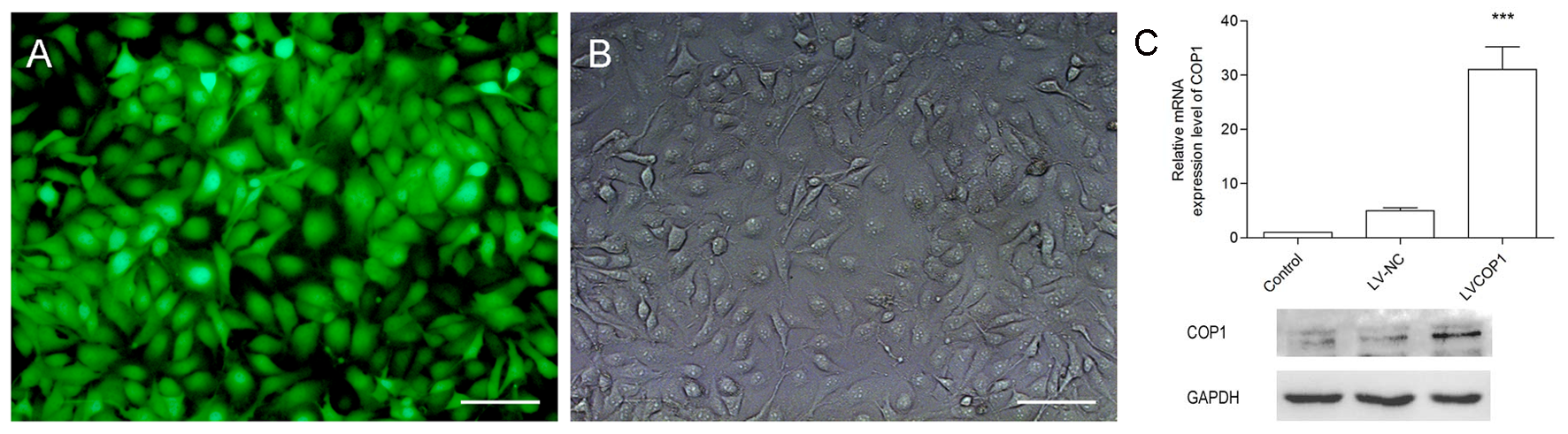

Supplement: Additional file 3: Figure S2. — Estimation of recombinant COP1 lentivirus (LVCOP1) infection efficiency in MDA-MB-231 cells. (A) LVCOP1 infected MDA-MB-231 was revealed by fluorescence microscopy via detecting the expression of GFP. (B) The same location of Figure 1A was observed under phase-contrast microscopy. (C) qRT-PCR and western blotting showed that COP1 expression was significantly increased in MDA-MB-231 cells infected with LVCOP1. Scale bar, 100 μm. (Students t tests, ***P < 0.001 versus MDA-MB-231 control group). [file 12885_2015_1151_MOESM3_ESM.tiff]

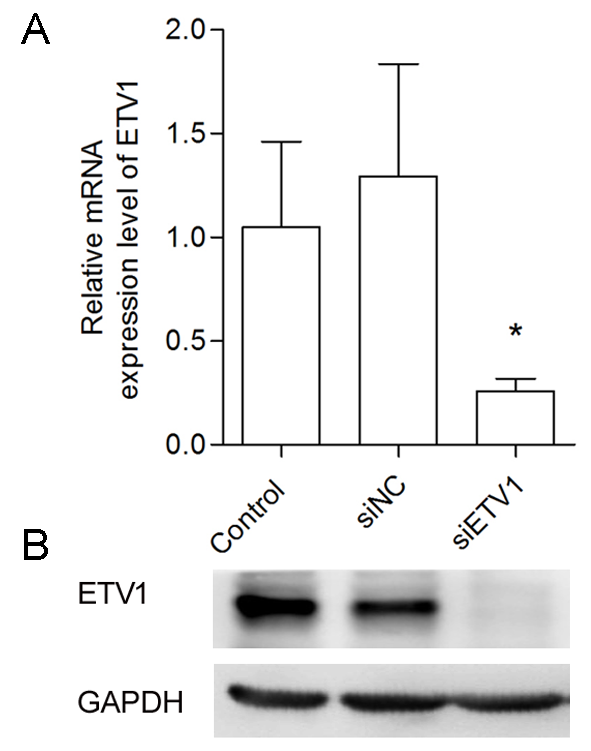

Supplement: Additional file 4: Figure S3. — Detection of the efficiency of ETV1 siRNA in MDA-MB-231 cells. Cells were transfected with siRNA against ETV1 or a non-specific RNA (siNC) as a negative control. The efficiency of siRNA was analyzed by qRT-PCR (A) and western blotting (B). Results showed that siETV1 could significantly decreased the expression of ETV1. (Students t tests, *P < 0.001 versus MDA-MB-231 control group). [file 12885_2015_1151_MOESM4_ESM.tiff]

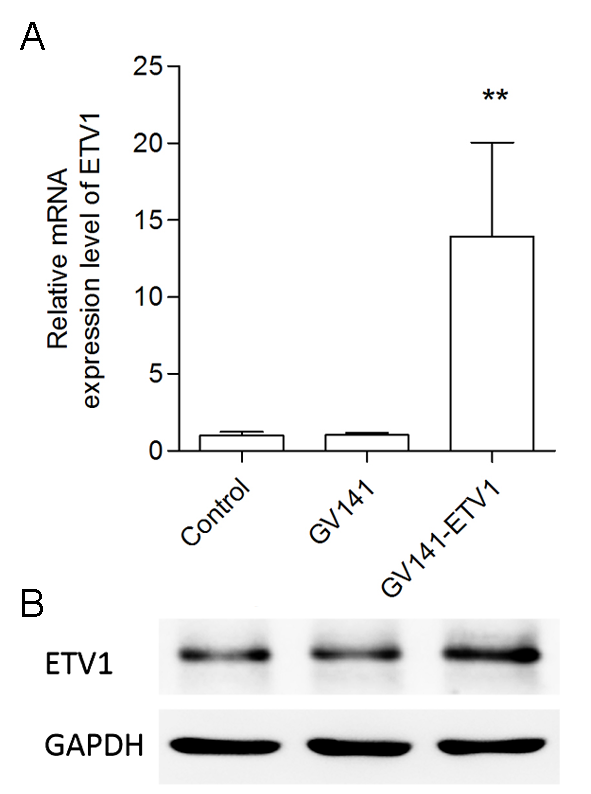

Supplement: Additional file 5: Figure S4. — Detection of the transfection efficiency of ETV1 expression vector in MDA-MB-231 cells. Full-length human ETV1 coding sequence was cloned into GV141 vector (GV141-ETV1). Cells were transfected with ETV1 expression vector or an empty GV141 vector. The efficiency of transfection was detected by qRT-PCR (A) and western blotting (B). Results showed that the expression of ETV1 was significantly up-regulated by GV141-ETV1. (Students t tests, **P < 0.05 versus control group). [file 12885_2015_1151_MOESM5_ESM.tiff]
