# Supplementary material for: COP1, the negative regulator of ETV1, influences prognosis in triple-negative breast cancer
Source: BMC Cancer. 2015 Mar 15;15:132. doi: 10.1186/s12885-015-1151-y (PMC4381371; doi:10.1186/s12885-015-1151-y)
Supplement: Additional file 2: Table S1. — siRNA sequences used in this article. The non-specific RNA (siNC) severed as a negative control are purchased from Ribobio (Ribobio Co., China). [file 12885_2015_1151_MOESM2_ESM.doc]

**Table S1.** **siRNA sequences used in this article.**

| Gene name | siRNA sequences |
| --- | --- |
| siETV1 | 5’ GGUCGAGGCAUGGAAUUUA dTdT 3’  3’ dTdT CCAGCUCCGUACCUUAAAU 5’ |
| siCOP1 | 5’ GGCUUAUACUCUCCUGUCA dTdT 3’  3’ dTdT CCGAAUAUGAGAGGACAGU 5’ |

The non-specific RNA (siNC) severed as a negative control are purchased from Ribobio (Ribobio Co., China).
