# Supplementary material for: COP1, the negative regulator of ETV1, influences prognosis in triple-negative breast cancer
Source: BMC Cancer. 2015 Mar 15;15:132. doi: 10.1186/s12885-015-1151-y (PMC4381371; doi:10.1186/s12885-015-1151-y)
Supplement: Additional file 6: Table S2. — Primer sequences used in this article. [file 12885_2015_1151_MOESM6_ESM.doc]

**Table S2. Primer sequences used in this article.**

| Gene name | Forward (5’-3’) | Reverse (5’-3’) |
| --- | --- | --- |
| ETV1 | CGCAGTCCATACCAGATAGCAGC | TGGCATCGTCGGCAAAGGAG |
| COP1 | ACTCTCCTGTCAGTGAGGATAGCACA | TGAGAACTGCCACTGAAACCTGGAG |
| β-actin | TGGCACCCAGCACAATGAA | CTAAGTCATAGTCCGCCTAGAAGCA |
